# A streamlined cohesin apparatus is sufficient for mitosis and meiosis in the protist *Tetrahymena*

**DOI:** 10.1007/s00412-018-0673-x

**Published:** 2018-06-12

**Authors:** Emine I. Ali, Josef Loidl, Rachel A. Howard-Till

**Affiliations:** Department of Chromosome Biology, Vienna Biocenter, University of Vienna, Vienna, Austria

**Keywords:** Meiosis, Mitosis, Cohesin, Chromosome maintenance

## Abstract

In order to understand its diverse functions, we have studied cohesin in the evolutionarily distant ciliate model organism *Tetrahymena thermophila*. In this binucleate cell, the heritable germline genome is maintained separately from the transcriptionally active somatic genome. In a previous study, we showed that a minimal cohesin complex in *Tetrahymena* consisted of homologs of Smc1, Smc3, and Rec8, which are present only in the germline nucleus, where they are needed for normal chromosome segregation as well as meiotic DNA repair. In this study, we confirm that a putative homolog of Scc3 is a member of this complex. In the absence of Scc3, Smc1 and Rec8 fail to localize to germline nuclei, Rec8 is hypo-phosphorylated, and cells show phenotypes similar to depletion of Smc1 and Rec8. We also identify a homolog of Scc2, which in other organisms is part of a heterodimeric complex (Scc2/Scc4) that helps load cohesin onto chromatin. In *Tetrahymena*, Scc2 interacts with Rec8 and Scc3, and its absence causes defects in mitotic and meiotic divisions. Scc2 is not required for chromosomal association of cohesin, but Rec8 is hypo-phosphorylated in its absence. Moreover, we did not identify a homolog of the cohesin loader Scc4, and no evidence was found of auxiliary factors, such as Eco1, Pds5, or WAPL. We propose that in *Tetrahymena*, a single, minimal cohesin complex performs all necessary functions for germline mitosis and meiosis, but is dispensable for transcription regulation and chromatin organization of the somatic genome.

**Electronic supplementary material:**

The online version of this article (10.1007/s00412-018-0673-x) contains supplementary material, which is available to authorized users.

## Introduction

The accurate segregation of chromosomes depends on an evolutionarily conserved multi-subunit complex known as cohesin. Cohesin is essential to keep sister chromatids together until the onset of anaphase (Nasmyth and Haering 2009). In addition to its function in cohesion, cohesin has been shown to be involved in genome organization, gene transcription, and DNA repair (Sjögren and Nasmyth 2001; Watrin and Peters 2006, 2009; Sjögren and Ström 2010; Seitan et al. 2011; Merkenschlager and Odom 2013). A now widely accepted model of genome organization and transcriptional regulation gives cohesin an active role in extruding chromatin loops. In interphase cells, the processivity of loop formation is regulated by the insulating factor CTCF, as well as cohesin modulating factors such as WAPL and Scc2/4, which all contribute to defining topologically associated domains (TADs) (Fudenberg et al. 2016; Haarhuis et al. 2017). TADs are thought to promote appropriate enhancer interactions with target genes, as well as discourage inappropriate interactions with genes in neighboring TADs, thus enforcing gene regulation networks (Smith et al. 2016; Poterlowicz et al. 2017). Cohesin also makes significant contributions to mitotic chromosome compaction in budding yeast (Schalbetter et al. 2017), which further emphasizes its organizational roles beyond cohesion.

The cohesin complex consists of four core components. Two structural maintenance of chromosome proteins, Smc1 and Smc3, interact with each other to form a V-shaped heterodimer. A ring-like structure forms when an α-kleisin protein (Scc1/Rad21/Rec8) binds to the Smc1-Smc3 dimer (Haering and Jessberger 2012; Remeseiro and Losada 2013; Gruber 2017). The fourth member of the complex, the HEAT repeat containing protein Scc3 in yeast (SA1/SA2 in humans), associates with the kleisin. Scc3 has been implicated in cohesin loading (Hu et al. 2011), maintenance of cohesion (Roig et al. 2014), and release from DNA (Hauf et al. 2005). The initial association of cohesin with chromatin takes place with the help of the Scc2-Scc4 (Nipbl-Mau2 in mammals) loader complex which stimulates ATP hydrolysis by the ATPase of the Smc1 and Smc3 head domains (Ciosk et al. 2000; Murayama and Uhlmann 2014).

**Fig. 1:**
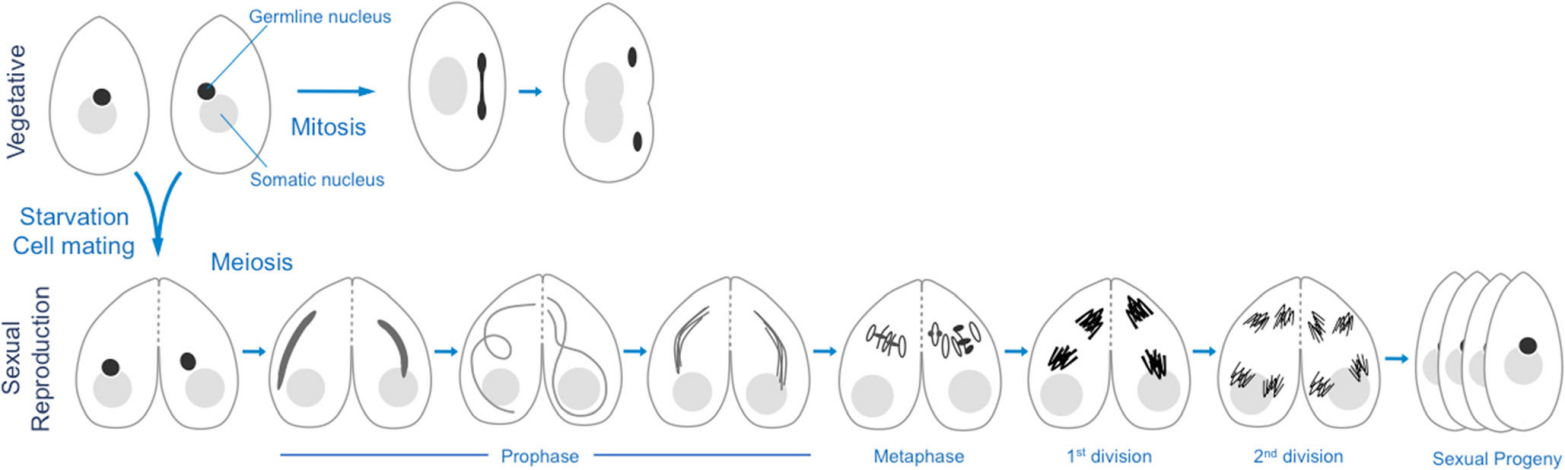
Life cycles of *Tetrahymena thermophila*. *Tetrahymena* cells have a transcriptionally active polyploid somatic nucleus and a transcriptionally silent diploid germline nucleus. *Tetrahymena* can reproduce by either vegetative or sexual reproduction. During vegetative growth, the germline nucleus undergoes mitosis, whereas the somatic nucleus undergoes an amitotic division where the chromosomes are divided roughly equally between the daughter cells. Under starvation conditions, two cells of different mating types can mate, and the germline nuclei of both cells divide by meiosis, whereas the somatic nuclei become degraded. During meiotic prophase, the germline nucleus elongates to form a crescent structure, in which homologous chromosomes pair and meiotic DSBs are formed and repaired by homologous recombination. Condensed bivalents are aligned at metaphase, which is followed by the first and second meiotic divisions. One meiotic product from each cell is selected for pronuclear exchange and fertilization, and the resulting zygotic nucleus divides twice to produce the new germline and somatic nuclei of the four sexual progeny

In order to gain insight into the evolutionary conservation of cohesin’s roles in chromosome dynamics, we have studied cohesin in the freshwater unicellular ciliate *Tetrahymena thermophila.* Each *Tetrahymena* cell contains two nuclei: a transcriptionally active, polyploid somatic nucleus and a transcriptionally silent, diploid germline nucleus (Karrer 2012). In a nutrient rich environment, *Tetrahymena* cells propagate by vegetative growth, during which the germline undergoes closed mitosis and the somatic nucleus divides roughly equally between two daughter cells by amitotic splitting (Fig. 1). Under starvation conditions, cells from different mating types pair and undergo sexual reproduction. In mating cultures, the germline nuclei undergo synchronous closed meiosis followed by reciprocal fertilization and post-zygotic mitoses to form new germline and somatic nuclei. The parental somatic nuclei are then degraded, and the new somatic nuclei undergo programmed genome rearrangements in which numerous transposon-like sequences are eliminated and the five germline chromosomes are fragmented to create ~225 minichromosomes (Noto and Mochizuki 2017). These somatic chromosomes range in size from about 20 kb to 3 Mb and are amplified to approximately 50 copies in mature cells (Hamilton et al. 2016).

Meiosis in *Tetrahymena* has several distinct features. The germline nuclei of the mating cells are in G2 when meiosis is initiated. During meiotic prophase, the germline nuclei elongate in response to meiotic DNA double-strand breaks (DSBs) (Fig. 1). At this stage, the centromeres and telomeres are attached to opposite ends of the highly elongated nuclei, creating an extreme bouquet arrangement. A synaptonemal complex (SC) is not formed; therefore, it is hypothesized that the elongated bouquet serves to align the chromosomes and promote homologous pairing and recombination (Loidl 2004; Mochizuki et al. 2008; Loidl et al. 2012). At the end of prophase, the germline nuclei shorten and condense to form distinct bivalents, then the meiotic divisions occur.

In *Tetrahymena*, Rec8 and Smc1 homologs are present only in the germline nucleus, and we showed previously that they are required for cohesion and the repair of DSBs (Howard-Till et al. 2013). Smc3 was identified by sequence homology and confirmed as an interacting partner of Rec8 by immunoprecipitation, defining a minimal core cohesin complex consisting of Smc1, Smc3, and Rec8. Here, we further characterize components of the cohesin complex and auxiliary factors.

## Results and discussion

### A homolog of Scc3 is part of the cohesin complex in *Tetrahymena*

**Fig. 2:**
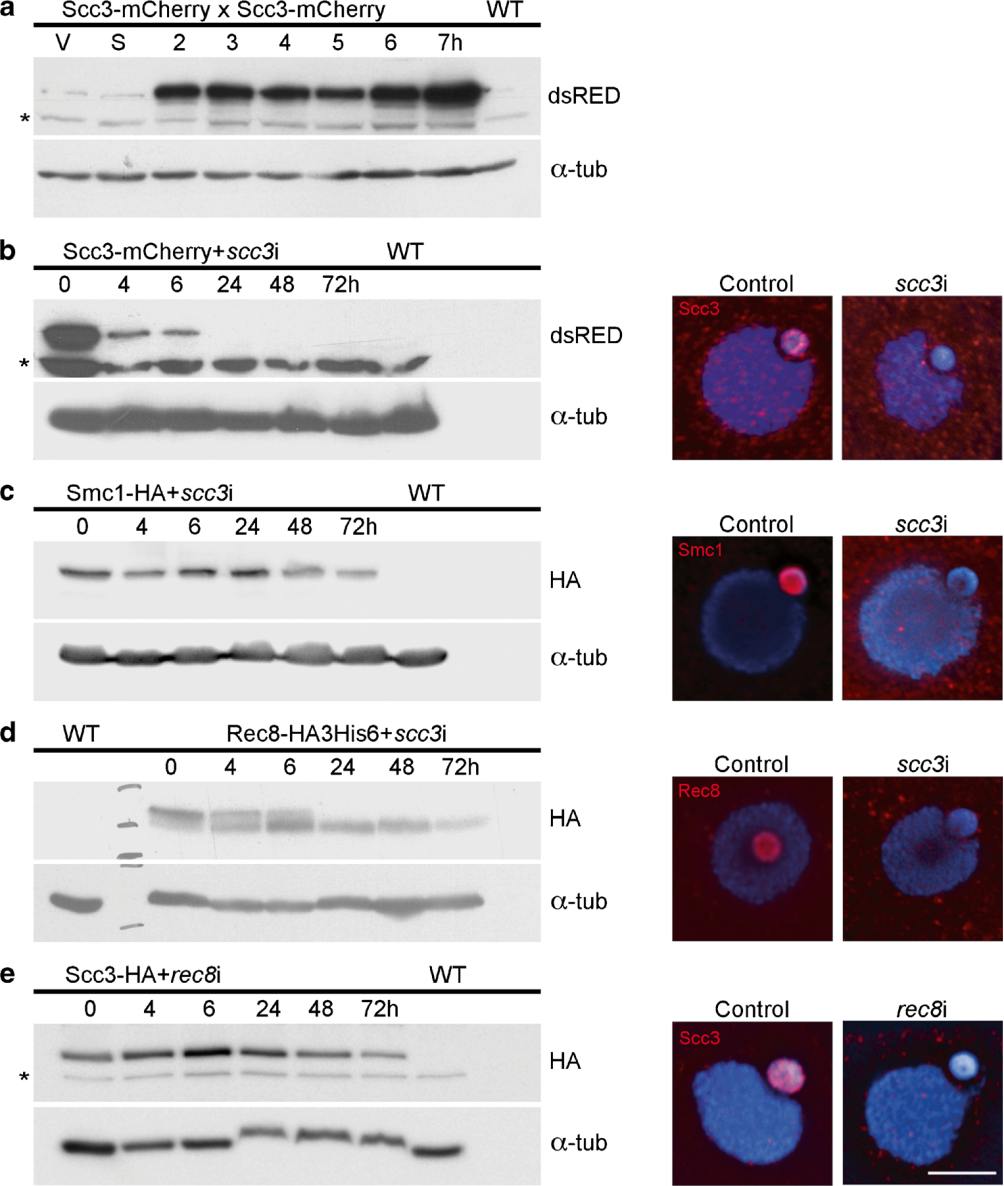
Localization of Smc1 and Rec8 is Scc3-dependent. **a** Scc3 is present throughout the cell cycle. Whole protein extracts were prepared from Scc3-mCherry expressing cells during vegetative growth (V), starvation (S), or 2–7 h after induction of mating, and blots were probed with anti-dsRed. **b** Complete depletion of Scc3 by RNAi is achieved by inducing RNAi for 24 h in vegetative cells. After 24 h of depletion by RNAi, Scc3-mCherry is not detected either by western blotting or in situ immunofluorescence. **c** Scc3 depletion in cells expressing Smc1-HA shows that Smc1 protein (as shown on the western blot) persists in the absence of Scc3. However, Smc1 is no longer visible in the germline nucleus. **d** Scc3 depletion caused the loss of a modified form of Rec8. Western blots of Rec8-HA3His6 show two forms of the protein; the upper band disappears upon Scc3 depletion. Immunofluorescence also shows a loss of Rec8 localization in *scc3i* cells. **e** Scc3 localization is dependent on Rec8. Protein levels of Scc3 do not change in the absence of Rec8, as shown by western blots. However, Scc3 no longer localizes to the germline nucleus, as seen by immunofluorescence. All tagged proteins were expressed from the endogenous loci. α-tubulin (α-tub) was used as a loading control in all western blots. Scale bar: 5 μm, asterisk indicates non-specific bands

A putative homolog of the HEAT repeat cohesin gene *SCC3* (TTHERM_00225630) was previously identified in the *Tetrahymena* genome (Howard-Till et al. 2013). The predicted protein has weak homology to the Scc3 of other organisms at the conserved STAG domain and showed a localization pattern identical to the other cohesin subunits (Howard-Till et al. 2013). Western blots of protein samples taken from cells expressing mCherry-tagged Scc3 from the endogenous locus show that the protein is present in both vegetative and meiotic cells (Fig. 2a). The higher relative abundance in meiosis may reflect the lack of synchrony of vegetative cells, where at any time only a small fraction of germline nuclei are in mitosis (average of 13% where 100 cells were counted in 3 vegetative samples). Immunofluorescence demonstrates the exclusive localization of Scc3 to the germline nucleus (Fig. 2b). To investigate whether *Tetrahymena* Scc3 is part of the cohesin complex, immunoprecipitation (IP) of Smc1-HA was performed from extracts of mating cells at 4 and 6 h after initiating mating, followed by mass spectrometry (MS) analysis. Scc3 was identified among the top Smc1-interacting proteins, together with the rest of the cohesin subunits (Table 1, Online Resource 1). A reciprocal experiment was also performed where Scc3-HA was immunoprecipitated, and all core cohesin subunits were identified as the best Scc3 interactors. These results confirm that the *Tetrahymena* Scc3 candidate is part of the cohesin complex. None of the IP experiments in either vegetative or meiotic cells identified additional cohesin or non-cohesin components (see Online Resource 1), confirming our previous assessment that *Tetrahymena* possesses a single cohesin complex that serves in both meiotic and mitotic chromosome segregation (Howard-Till et al. 2013).

**Table 1:** Mass spectrometry results for Smc1-HA and Scc3-HA immunoprecipitation

Bait protein	Proteins identified	Unique peptides	Sequence coverage (%)
Smc1-HA			
	Smc1	116	71.1
	Smc3	93	55.8
	Scc3	80	54
	Rec8	36	59.5
Scc3-HA			
	Scc3	74	48.2
	Smc1	79	55.4
	Smc3	64	43.5
	Rec8	27	41.5

### Scc3 is required for localization and chromatin association of Smc1 and Rec8

To investigate the function of Scc3 in *Tetrahymena*, the protein was depleted by inducible RNA interference (RNAi). Induction of Scc3 RNAi (in the following, *scc3*i for short) for 24 h is sufficient for complete depletion of the protein (Fig. 2b). In order to determine its effect on other cohesin subunits, *scc3*i was performed in cells expressing either Smc1-HA or Rec8-HA3His6. Depletion of Scc3 had no effect on Smc1 protein level but it did prevent its localization to the germline nucleus (Fig. 2c). Scc3 was also required for Rec8 localization to the germline nucleus (Fig. 2d). These results are consistent with experiments in mice or budding yeast that have shown Scc3 is required for the localization of cohesin on chromatin (Fukuda et al. 2014; Roig et al. 2014). Although cohesin localization is generally Scc3 dependent, the requirement of Scc3 for the stability of cohesin proteins varies among different models. In mouse spermatocytes, reduction of Scc3/STAG3 results in reduced stability of Rec8, but not Smc1 or Smc3 (Fukuda et al. 2014). In budding yeast, however, degron-based inactivation of Scc3 does not affect levels of Scc1 or Smc1 (Roig et al. 2014).

**Fig. 3:**
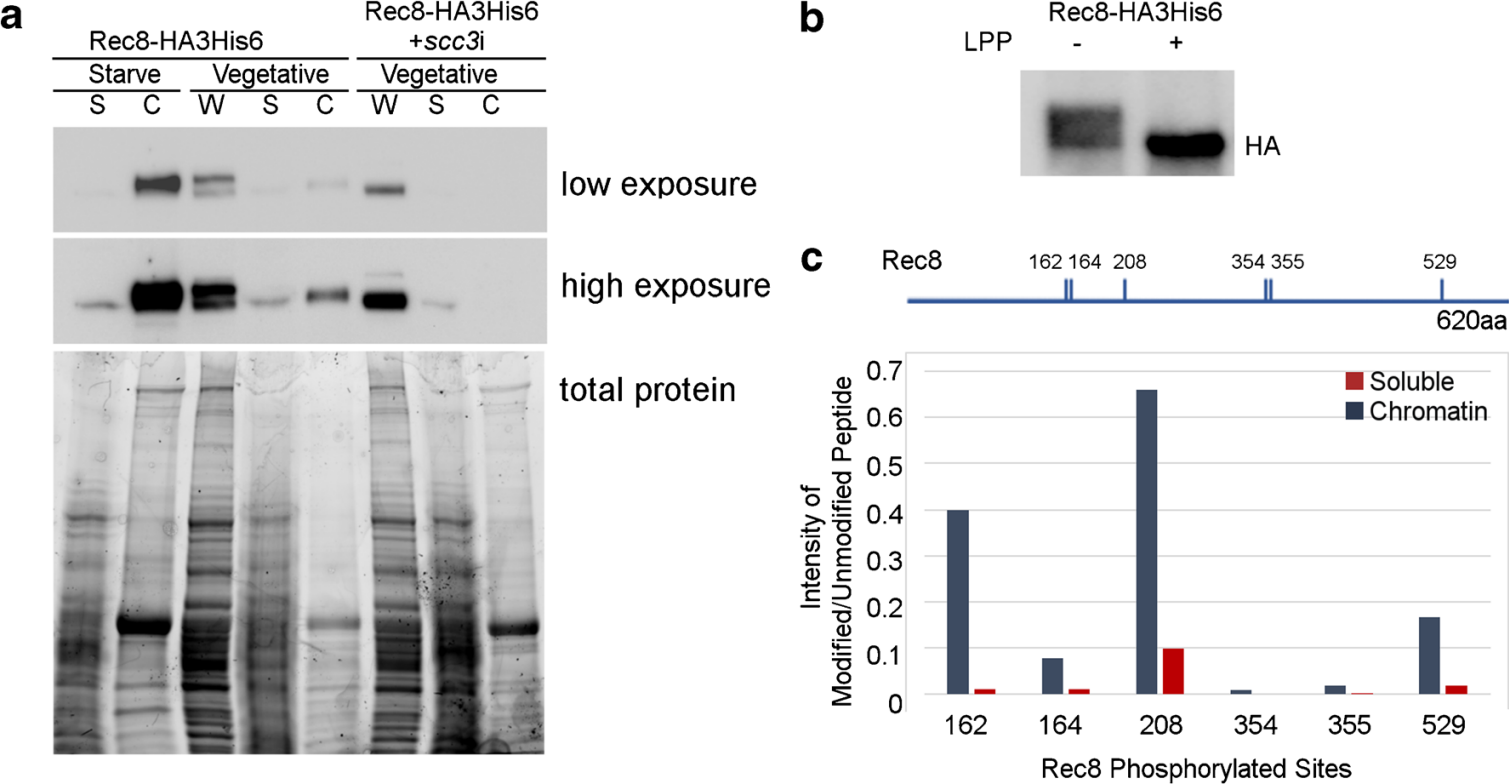
Chromatin association and phosphorylation of Rec8 is Scc3-dependent. **a** Cellular fractionation of starved and vegetative cells expressing Rec8-HAHis6 shows that the chromatin bound form is the slower migrating form. W: whole cell lysate, S: soluble fraction, C: chromatin bound fraction. In *scc3*i cells, Rec8 is not detected in the chromatin fraction, and only the faster migrating form is present in whole cells and the soluble fraction. Total protein loading is visualized in the Bio-Rad stain free gel prior to blotting. **b** Chromatin bound Rec8 is phosphorylated. Rec8-HAHis6 immunoprecipitated from the chromatin fraction of starved cells was treated with Lambda protein phosphatase (LPP). Both forms are present in the mock treated sample, but the phosphatase treated sample contains only the faster migrating form, indicating the modified form is phosphorylated. **c** Immunoprecipitation experiments were performed on soluble and chromatin fractions of Rec8-HA3His6 vegetative cells. The phosphorylated sites on Rec8 were identified by mass spectrometry analysis. Six residues show increased levels of phosphorylation in the chromatin fraction compared to the soluble fraction. The graphed values represent the ratio of the intensity of modified peptide to the intensity of unmodified counter peptide. Blue bars: chromatin fraction, Red bars: soluble fraction

Although depletion of Scc3 did not affect protein levels of Rec8, it did alter its modification. Western blots of Rec8 show a double band, with the upper band likely representing phosphorylated Rec8 (see below). Depletion of Scc3 results in the loss of the slower migrating form (Fig. 2d). Cell fractionation experiments show that the soluble fraction of cohesin contains only the faster migrating form, whereas the chromatin-bound fraction contains the modified form. Cells depleted of Scc3 had no detectible Rec8 present in the chromatin bound fraction (Fig. 3a). To determine if the upper band consisted of phosphorylated Rec8, IPs from the chromatin fraction of starved cells were treated with lambda protein phosphatase. In the mock treatment, both forms of Rec8 are present, but in the phosphatase treated sample, only the lower band remains, indicating that the slower migrating form is phosphorylated (Fig. 3b). To determine which residues were phosphorylated on chromatin bound Rec8, we subjected the IPs from the chromatin and soluble fraction of vegetative Rec8-HA3His6 samples to MS analysis (Fig. 3c, Online Resource 1). Six residues, Serine 162, 164, 208, 354, 355, and 529, were identified as phosphorylated. All of these showed at least sixfold higher phosphorylation in the chromatin-bound fraction (Fig. 3c). These results suggest that in the absence of Scc3, Rec8 is hypo-phosphorylated, and coincidentally, cohesin fails to localize to germline chromatin. At present, it is difficult to determine the cause and effect relationship of these events. In the future, it will be important to create phospho-mutants of these residues to determine their importance in the loading or binding stability of cohesin.

**Fig. 4:**
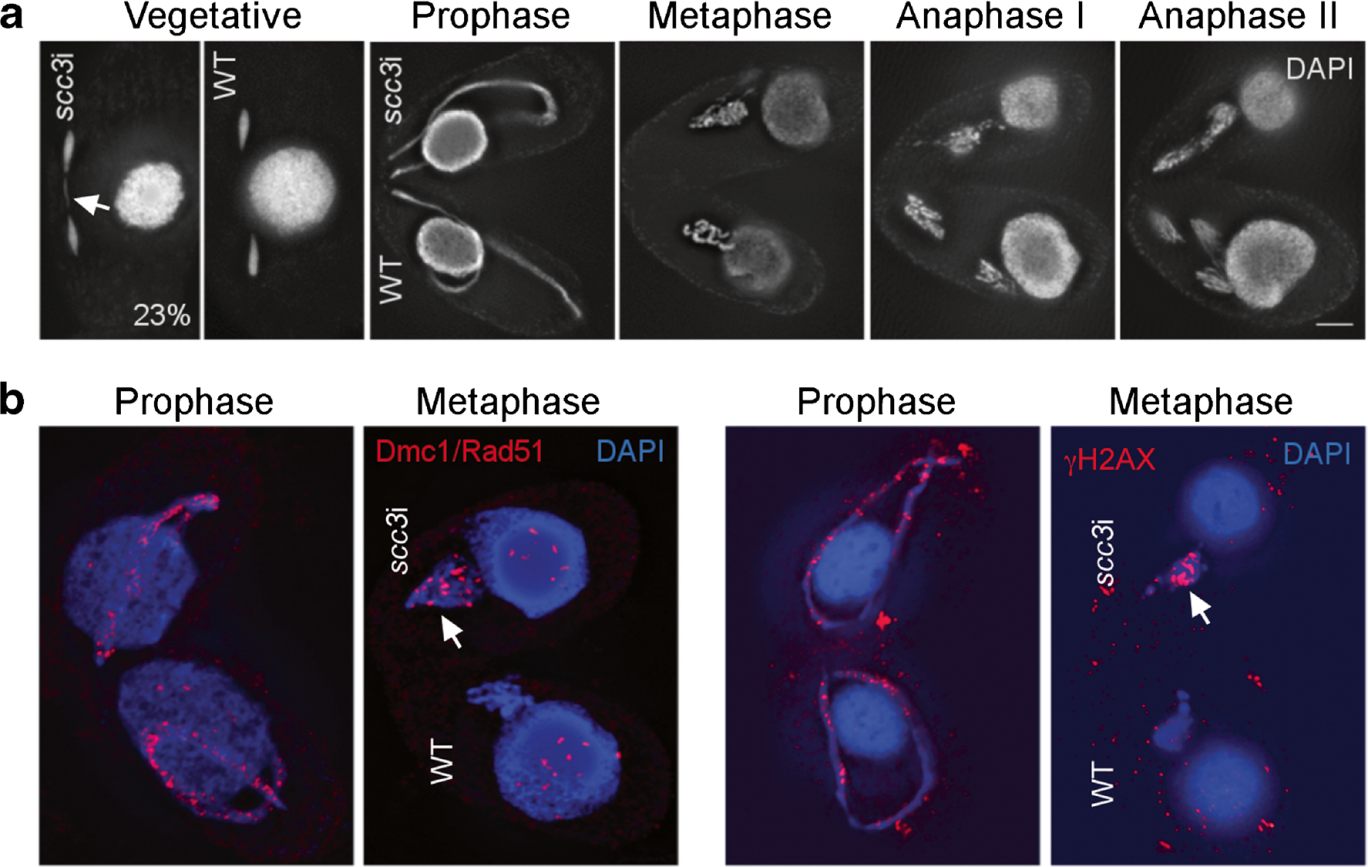
Depletion of Scc3 causes meiotic arrest with unrepaired DSBs. **a** Scc3 RNAi was induced in growing cells for 24 h to fully deplete the protein. Vegetative cell shows a mitotic division with lagging chromosomes (arrow). A WT mitotic cell in anaphase is shown for reference. Starved *scc3*i cells were mated with WT cells. Prophase occurs normally for both cells, then the *scc3*i partner (top cell) arrests in a metaphase-like state, while the WT partner (bottom cell) completes meiotic divisions. **b** DSBs persist in *scc*3i cells. Mating cells were fixed in high detergent and stained for Dmc1/Rad51 (left) or by modified Schaudinn’s fixation and stained for γ-H2A.X (right). Dmc1/Rad51 and γ-H2A.X foci are markers for unrepaired DSBs. In the WT partner, DSBs are only detected in prophase stages of meiosis. However, the *scc3*i cells mated with WT cells show persistent Dmc1/Rad51 and γ-H2A.X foci in the arrested *scc3*i cells at metaphase (arrows in top cells). Scale bars: 5 μm

Depletion of Scc3 resulted in chromosomal defects similar to those found for Rec8 or Smc1 depletion (Howard-Till et al. 2013). In vegetatively growing cells, 23% of *scc3*i cells in mitotic anaphase showed lagging chromosomes or bridged anaphases (Fig. 4a), whereas controls showed no bridging (100 anaphases counted for each). Mating cells arrested prior to performing meiotic divisions. Meiotic prophase occurred normally, but condensed bivalent chromosomes never formed, and cells arrested with uncondensed germline chromatin (Fig. 4a). Germline chromosomes showed signs of unrepaired DSBs, as assayed by immunostaining for the DSB markers Dmc1/Rad51 and phosphorylated γH2A.X (Fig. 4b).

The DSB repair defect may be due to a failure to form chromosome axes. Cohesin has been identified as a part of meiotic chromosome axes (Klein et al. 1999; Novak et al. 2008) and is required for SC assembly (Hopkins et al. 2014; Fukuda et al. 2014; Ishiguro and Watanabe 2016). Rec8 is also a key component of linear elements in *Schizosaccharomyces pombe*, which does not have a canonical SC structure (Molnar et al. 1995; Loidl 2006; Ding et al. 2016). Although *Tetrahymena* does not utilize a SC, it is likely that cohesin is important to establish a chromosomal axis to support meiotic pairing and recombination. New cohesin that loads during prophase may participate in chromosome reorganization while the germline nucleus elongates and meiotic recombination occurs.

**Fig. 5:**
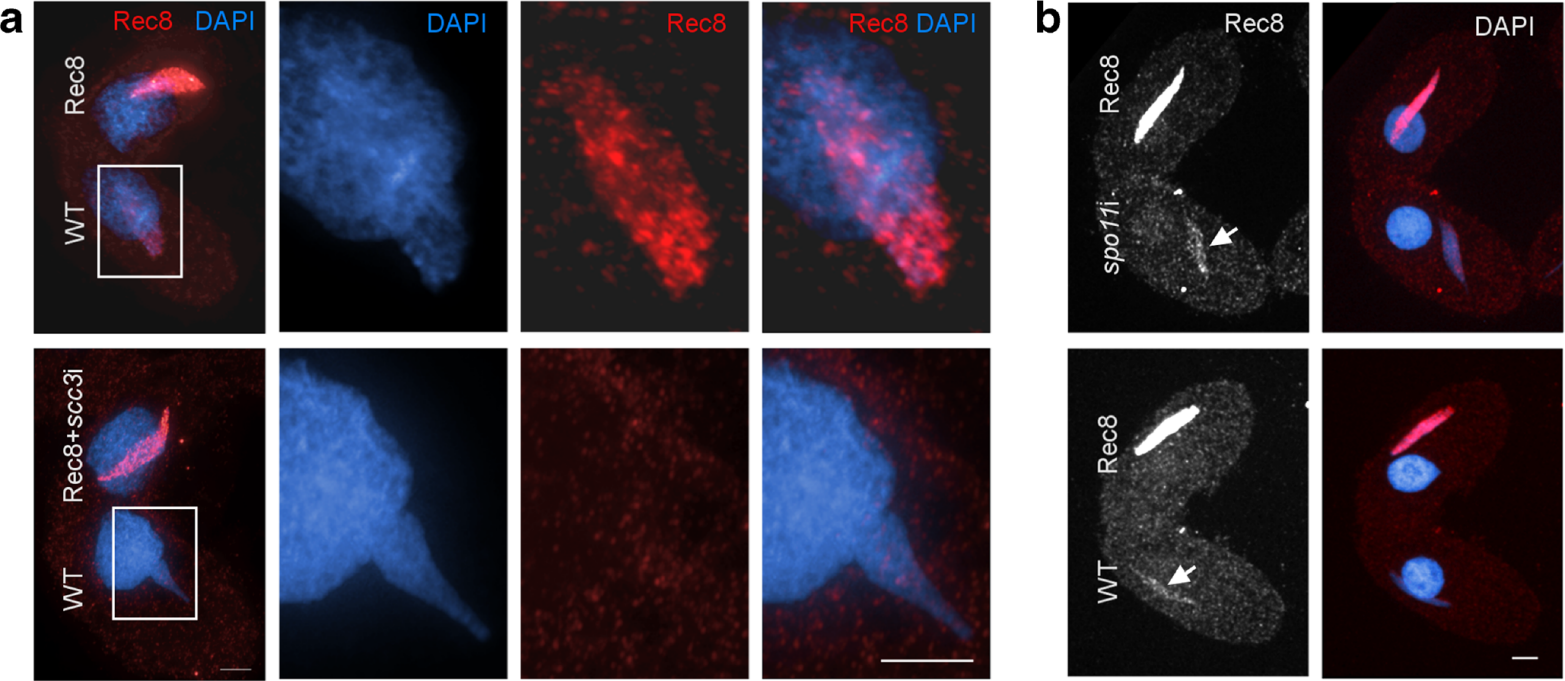
Rec8 loading is Scc3-dependent, but Spo11-independent. **a** Depletion of Scc3 prevents loading of Rec8 to meiotic prophase chromatin. WT cells were mated with Rec8-mCherry cells not depleted or depleted of Scc3. 4 h after initiating mating, cells were fixed in high-detergent. Enlarged images (boxes) show the germline nucleus of the WT cell. Rec8 signal was detected in the WT partner of the control sample, indicating that transferred Rec8 is loading onto chromatin during prophase (top panels). However, depletion of Scc3 abolished the loading of transferred Rec8 in the WT cell (bottom panels). **b** Meiotic loading of cohesin is not dependent on DSBs. Rec8-HA3His6 cells were mated with either WT cells or *spo11*i cells. The localization of Rec8 to the WT partner (arrows) is not changed in the absence of Spo11. Scale bars: 5 μm

We then tested whether depletion of Scc3 affected cohesin loading during meiosis. When cells expressing tagged Smc1 or Rec8 were mated to wild-type (WT) cells, a small amount of the tagged cohesin could be detected in the germline nucleus of the untagged cell in late prophase (Fig. 5a). This is due to the ability of mating cells to exchange proteins (McDonald 1966). Although this localization occurs at the time of meiotic DSB repair, we found it is not dependent on the induction of DSBs by Spo11 (Fig. 5b). When *scc3*i was induced specifically during meiosis, the localization of the tagged cohesin in the WT cell could not be detected, despite the presence of abundant tagged cohesin in the other cell (Fig. 5a). This suggests that expression of Scc3 during meiosis is required to load cohesin at that time, and implies that any Scc3 present on chromosomes prior to mating is already stably associated with bound cohesin complexes and is not able to participate in loading new cohesin.

Experiments in budding yeast indicate that Scc3 is required for loading cohesin onto chromosomes, as well as maintaining cohesion (Roig et al. 2014). Structural and biochemical analysis of Psc3 (Scc3) in *S. pombe* indicates that it interacts with the Scc2/Scc4 loading complex, and mutations affecting this interaction reduced loading efficiency (Murayama and Uhlmann 2014). Similarly, IP experiments in budding yeast indicate a direct interaction between Scc3 and Scc2/Scc4 (Orgil et al. 2015). While *Tetrahymena* Scc3 is important for loading, the mechanism may be different, because of the deviating function of Scc2 (see below). Overall, data from this and other studies indicate that Scc3 is an essential component of the cohesin complex and not merely an auxiliary factor.

### A homolog of the cohesin loading component Scc2/NipBL localizes to the germline nucleus and associates with cohesin proteins

**Fig. 6:**
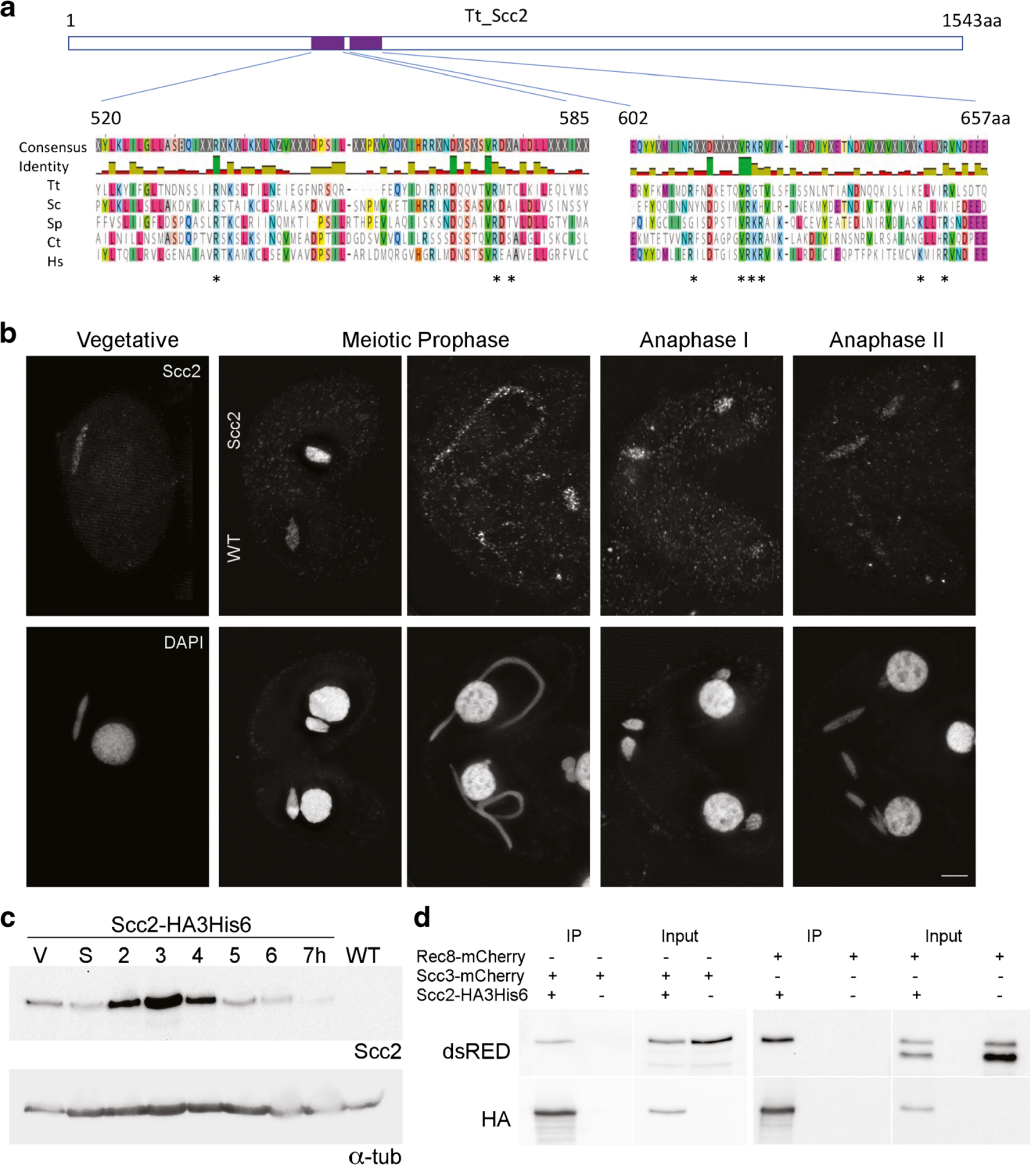
A *Tetrahymena* Scc2 homolog interacts with Rec8 and Scc3 and localizes to the germline nucleus. **a** *Tetrahymena* TTHERM_00678460 (Scc2) shows homology to Scc2 of other species. Alignments were produced using Scc2 protein sequences from 5 species, Tt: *Tetrahymena thermophila*, Sc: *Saccharomyces cerevisiae, Sp: Schizosaccharomyces pombe*, Ct: *Chaetomium thermophilum*, and Hs: *Homo sapiens.* Shown are the regions of highest homology, which occur around sites that have been identified in other organisms as important for interaction with the kleisin Scc1 (indicated by asterisk). Alignment was created with the Muscle algorithm 3.8.425 in Geneious software version 11.0.4 (Edgar 2004; Kearse et al. 2012). **b** Scc2 is present in the germline nucleus at all growth stages and appears most abundant in early meiotic prophase. **c** The protein level of Scc2 increases during early conjugation. Protein extracts were prepared from vegetative (V), starved (S), or mating cells (2–7 h after induction of mating) expressing Scc2-HA3His6. Loading control: α-tubulin. **d** Scc2-HA3His6 was immunoprecipitated from cells co-expressing either Rec8-mCherry or Scc3-mCherry. Scc3 and one form of Rec8 co-precipitated with Scc2. IPs were performed using lysates prepared from meiotic cells 4 h after mating induction. All tagged proteins were expressed from the endogenous loci. Scale bar: 5 μm

Using standard homology searches, we have been unable to identify *Tetrahymena* homologs of other proteins known to interact with cohesin, such as the cohesin loading complex Scc2/Scc4 or the stabilizing/destabilizing factors Eco1, Pds5 and WAPL. However, TTHERM_00678460 was identified as a potential homolog of the Scc2/NipBL cohesin loader from a screen of meiotically upregulated candidate genes: it shows increased gene expression 2–4 h after initiating mating (http://tfgd.ihb.ac.cn/). Alignment of the TTHERM_00678460 predicted protein sequence with Scc2 homologs from other organisms shows good homology in regions that are important for interaction with the kleisin Scc1 (Kikuchi et al. 2016) (Fig. 6a).

Strains that expressed Scc2-HA3His6 from the endogenous locus were produced and used to evaluate localization and protein levels throughout the *Tetrahymena* life cycle. Immuno-fluorescence showed localization of Scc2 in the germline nucleus of both vegetative and mating cells. The Scc2-HA3His6 signal was most intense in early prophase of meiosis, as the germline nucleus was elongating, and decreased in later stages of meiosis (Fig. 6b, Fig. S1). High-detergent fixations were also performed (Fig. S1). Under such conditions, most proteins not stably bound to chromatin are washed out of the nucleus (Howard-Till et al. 2011). Unlike Rec8, which shows strong binding to chromatin throughout the cell cycle, Scc2-HA3His6 showed chromatin association primarily in early meiotic prophase (Fig. S1). Western blot analysis also showed Scc2 levels were high during early mating time points, and decreased after completion of meiosis (Fig. 6c).

To determine if Scc2 interacted with cohesin proteins, strains were produced which co-expressed Scc2-HA3His6 and either Rec8-mCherry or Scc3-mCherry. Co-immunoprecipitation (Co-IP) experiments showed that both Rec8 and Scc3 interact with Scc2-HA3His6 (Fig. 6d). IP samples subjected to MS were unable to identify additional interacting partners (Online Resource 1). Notable in its absence was an Scc4 homolog, which is normally found complexed with Scc2. However, the homology of TTHERM_00678460 with other Scc2 proteins, its localization, and its interaction with Smc1 and Scc3 supported its identity as the genuine Scc2 ortholog.

Scc2 plays a critical role in regulating cohesin function in many organisms. This is especially the case in vertebrates, where even modest reductions of Scc2 expression can cause severe defects. The majority of cases of the human cohesinopathy, Cornelia de Lange Syndrome (CdLS), are caused by heterozygous mutations in Scc2, and in one case, CdLS was caused by a reduction in expression of only 15% (Borck et al. 2006; Rohatgi et al. 2010). These statistics indicate that cells are very sensitive to the levels of this protein.

### *Tetrahymena* Scc2 is required for chromosome segregation in mitosis and DSB repair in meiosis

**Fig. 7:**
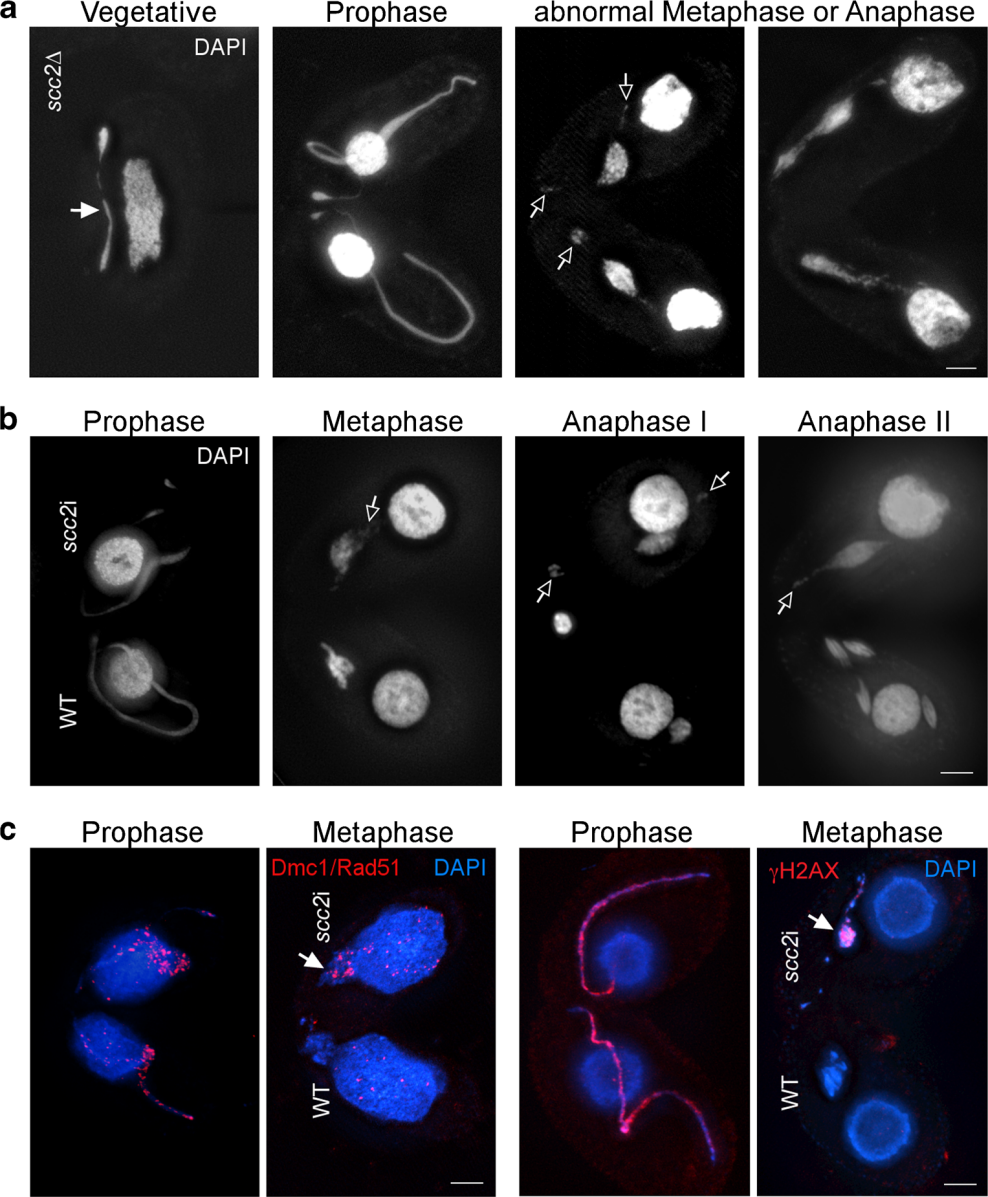
Lack of Scc2 causes defective chromosome segregation and meiotic arrest with unrepaired DSBs. **a** Vegetative *scc2*∆ cells show mitotic bridging (filled arrow). Mating cells arrest with uncondensed chromatin at abnormal metaphase or anaphase. Open arrows indicate DAPI bodies that appear to be centromere-containing fragments that are detached from the rest of chromosomes and migrate to the poles. **b** In matings between *scc2*i and WT cells, the *scc2*i partner arrests while the WT partner completes meiotic divisions. Open arrows indicate possible chromosome fragments. **c** Staining for DSB markers shows that arrested *scc2*i cells do not repair meiotic DSBs. In meiotic *scc2*i cells, Dmc1/Rad51 (left) and gH2AX foci (right) are still present at metaphase (arrows), indicating the presence of unrepaired DSBs. Scale bars: 5 μm

To determine if Scc2 has a role in cohesion or chromosome segregation in *Tetrahymena*, knockout strains were produced in which part of the *SCC2* gene was deleted from all somatic chromosome copies using co-deletion. (This method utilizes the natural *Tetrahymena* phenomenon of genome diminution (Hayashi and Mochizuki 2015).) Overall, the phenotype of these cells was similar to cells depleted of cohesin components (Howard-Till et al. 2013). Vegetatively growing cells showed defective chromosome segregation in mitosis (Fig. 7a), and germline nuclei changed size over time (Fig. S2), indicating a loss or gain of chromosomes. Mating cells were unable to complete meiosis and arrested prior to anaphase (Fig. 7a). Arrested cells showed cytological markers consistent with unrepaired DSBs: Dmc1/Rad51 and γ-H2A.X foci were still present in arrested cells at stages where DNA repair was complete in the WT (Fig. 7c). To exclude the possibility that defective mitotic divisions were affecting subsequent meiosis, RNAi strains were produced in order to selectively deplete Scc2 from meiotic cells. *scc2*i strains showed similar meiotic phenotypes to the knockout strains (Fig. 7b).

**Fig. 8:**
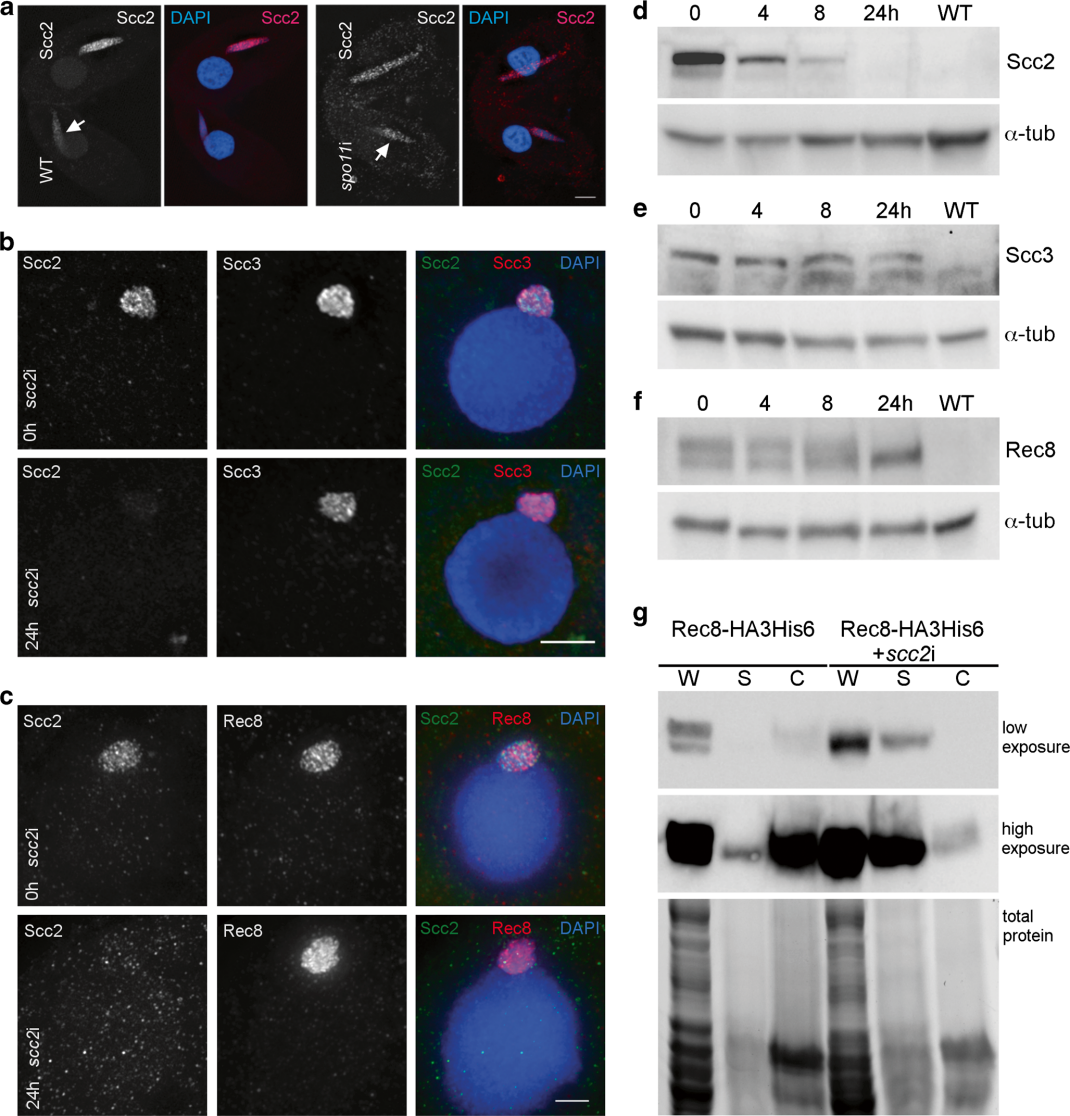
Scc2 localization is not DSB dependent, and cohesin containing hypo-phosphorylated Rec8 associates with chromatin in the absence of Scc2. **a** Spo11-induced DSBs are not required for Scc2 localization. Scc2-HA3His6 cells were mated with WT cells or *spo11i* induced cells and fixed 4 h after initiating mating. Localization of transferred Scc2 to the WT nucleus (arrows) occurs in the absence of Spo11. **b** Scc3 localizes to chromatin in the absence of Scc2. *scc2i* cells expressing Scc3-mCherry and Scc2-HA3His6 were fixed in high-detergent at 0 h and 24 h after induction of RNAi. **c** Rec8 localizes to chromatin in the absence of Scc2. *scc2i* cells expressing Rec8-mCherry and Scc2-HA3His6 were fixed in high-detergent at 0 h and 24 h after induction of RNAi. **d**–**f** Western analysis of Scc2, Scc3, and Rec8 after depletion of Scc2. **d** Scc2-HA3His6 + *scc2*i cells show complete loss of Scc2 after 24 h of RNAi induction **e** Scc3 levels do not change after *scc2i* in cells expressing Scc3-mCherry. **f** Rec8 appears hypo-phosphorylated after 24-h induction of *scc2*i in cells expressing Rec8-HA3His6. All western blots were run with total cell extracts prepared at the time indicated after induction of RNAi. α-tubulin is shown as a loading control. **g** Cellular fractionation of uninduced and induced *scc2*i cells expressing Rec8-HA3His6 shows Rec8 is still found in the chromatin fraction. Whole cell lysates from the RNAi cells only show the faster migrating form, indicating that Rec8 is hypo-phosphorylated. W: whole cell lysate, S: soluble fraction, C: chromatin bound fraction. Total protein is visualized in the Bio-Rad stain free gel prior to blotting. Scale bars: 5 μm

To further investigate the link between Scc2 and DSB repair, we tested if Scc2 localization on meiotic chromosomes was dependent on DSBs. *spo11*i cells were mated with Scc2-HA3His6 cells. Scc2 localization appeared unchanged in matings with *spo11*i cells, as compared to matings with WT cells (Fig. 8a). This indicates that Scc2 localization on prophase chromosomes is not dependent on DSBs. It is possible that its presence is instead in preparation for chromatin remodeling in advance of meiotic recombination, rather than in response to DSBs. Scc2 stimulates ATP hydrolysis, which has been shown to be required for translocation of cohesin on DNA, as well as cohesin loading and acetylation (Murayama and Uhlmann 2014; Ladurner et al. 2014; Elbatsh et al. 2016; Kanke et al. 2016). Cohesin’s mechanism for loop extrusion may be related to translocation, in which case Scc2 would ultimately be a stimulator of loop extrusion (Sanborn et al. 2015; Fudenberg et al. 2016; Davidson et al. 2016). Looping models have also been proposed for the organization of the axes of pairing chromosomes (axial elements) (Kleckner 2006), and axial elements are essential for meiotic recombination (Klein et al. 1999; Storlazzi et al. 2008; Panizza et al. 2011). At this time, meiotic axis structures in *Tetrahymena* are undefined; therefore, we are unable to determine a link between Scc2 and axis formation.

### Scc2 is not required for cohesin localization, but cohesin is required for the localization of Scc2

Scc2 is primarily known for its role in loading cohesin, as part of the Scc2/Scc4 loading complex (Ciosk et al. 2000; Kogut et al. 2009; Bermudez et al. 2012; Chao et al. 2015). To test the requirement of Scc2 for cohesin localization and chromosomal association, Scc2 was depleted by RNAi, which resulted in the apparently complete loss of the protein within 24 h (Fig. 8d). When Scc2 was depleted from *Tetrahymena* cells expressing tagged Rec8 or Scc3, the cohesin proteins still localized to the germline nucleus, and their persistence after high-detergent fixation indicated they still associated with chromatin (Fig. 8b, c, Fig. S3). This result is in contrast to organisms such as budding yeast and *C. elegans*, where association of cohesin with chromosomes is dependent on Scc2 (Ciosk et al. 2000; Lightfoot et al. 2011). Western blot analysis of *scc2*i cells expressing Rec8-HA3His6 or Scc3-mCherry shows that levels of the cohesin subunits do not change substantially after depletion of Scc2 (Fig. 8e, f). However, similar to the case in *scc3*i cells, the phosphorylation of Rec8 appears to be reduced (Fig. 8f). In contrast to the *scc3*i situation, cell fractionation experiments performed on *scc2*i + Rec8-HA3His6 cells show some hypo-phosphorylated Rec8 present in the chromatin fraction (Fig. 8g). Phosphorylation of Rec8 may therefore reflect a functional state of cohesin, and not merely chromosomal association.

To determine if the segregation problems occurring during mitosis were due to loss of cohesion, we assessed cohesion in *scc2*i strains. Cohesion was tested by evaluation of FISH (Fluorescence in situ hybridization) signals in meiotic prophase nuclei, which normally appear as 1 (paired) or 2 dots (unpaired) (Fig. S3a). (Due to the condensed state of the germline nucleus in vegetative growth, we are unable to obtain reliable FISH hybridization in mitotic cells.) The presence of 3 or 4 spots indicates the separation of sister chromatids by loss of cohesion, as was the case in 42% of nuclei after depletion of Rec8 (Howard-Till et al. 2013). FISH analysis was performed in 4 independent matings of WT and *scc2*i strains with and without induction of RNAi. Uninduced *scc2*i strains showed an average of 16% of nuclei with 3 or 4 spots, which increased to 26% in induced cells, compared to 10% in WT strains (Fig. S3c). The difference between WT and induced *scc2*i cells was statistically significant (Mann-Whitney test, *p* = .0286), but the difference between uninduced and induced scc2i cells was not. Although depletion of Scc2 appears to be complete by western blotting (Fig. S3b), it is possible an undetectable amount of residual protein is enough to allow sufficient loading or activation of cohesin to achieve almost normal levels of cohesion. Indeed, studies in yeast have shown that very little cohesin is needed to achieve cohesion (Heidinger-Pauli et al. 2010). Unfortunately, due to the stochastic gain and loss of chromosomes in the scc2∆ strain, we were unable to analyze cohesion in the deletion background.

**Fig. 9:**
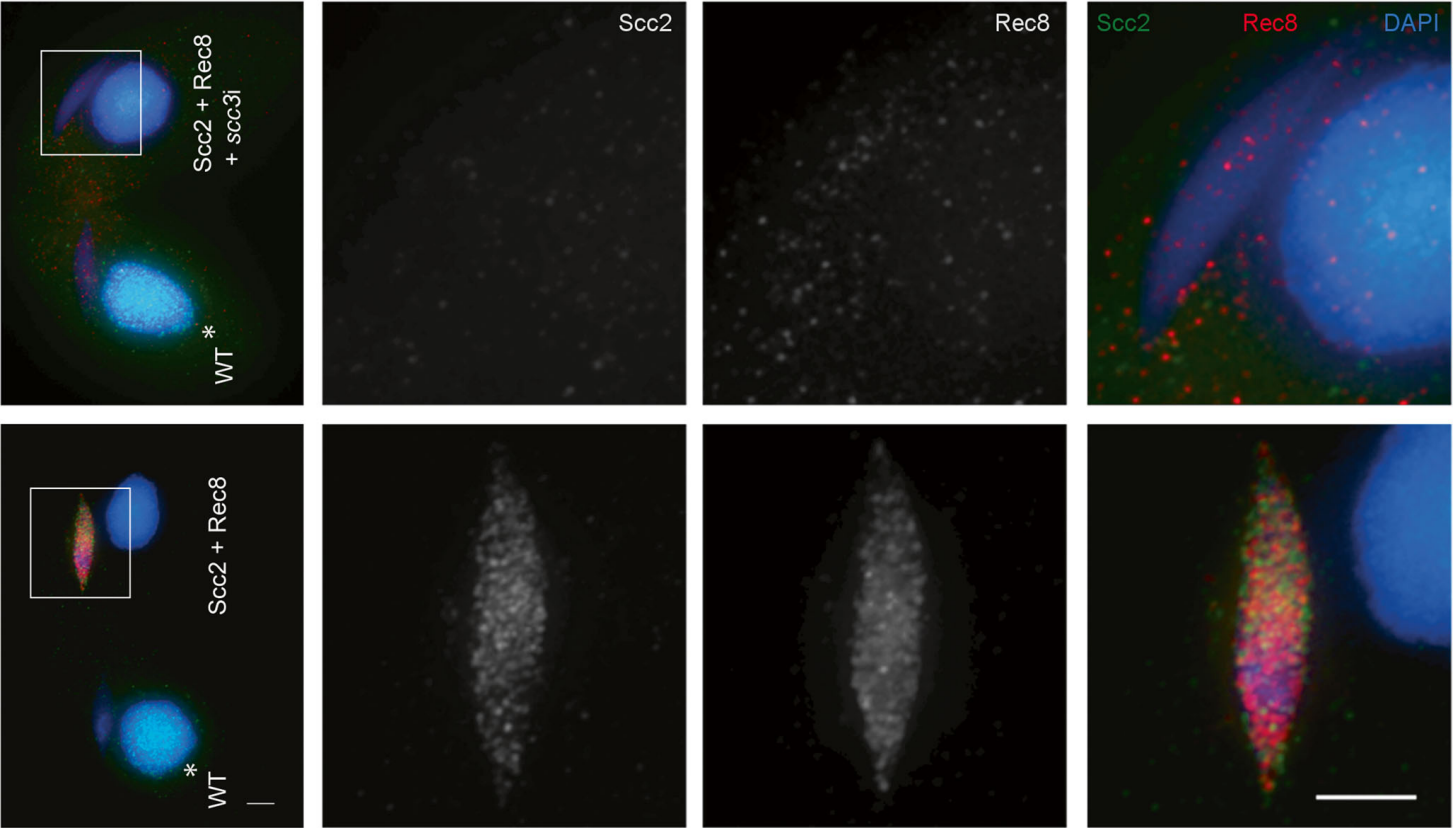
Depletion of Scc3 results in loss of Rec8 and Scc2 localization. *scc3*i or control cells expressing Rec8-mCherry and Scc2-HA3His6 were fixed 3 h after initiating mating and co-stained with antibodies against HA and dsRed. WT cells have HA signal in the somatic nucleus (*) as a marker. Scale bars: 5 μm

We next tested if the localization of Scc2 depended on the presence of cohesin. Cohesin was depleted from cells expressing Scc2-HA3His6 and Rec8-mCherry by 24-h induction of *scc3*i, which resulted in the complete loss of Rec8 localization, as well as that of Scc2 (Fig. 9). This suggests that localization of Scc2 is cohesin dependent, similar to the finding in budding yeast that association of Scc2 with centromeres is dependent on the presence of Scc1 (Fernius et al. 2013). A partial dependence on cohesin for Scc2 association with DNA was also demonstrated in HELA cells (Fernius et al. 2013; Rhodes et al. 2017). However, Scc2 in other organisms also has a DNA binding activity independent of cohesin, and it has been reported to associate with gene promoters (Murayama and Uhlmann 2014; Zuin et al. 2014; Rhodes et al. 2017). In *Tetrahymena* cells, cohesin and Scc2 are found only in the germline nucleus, which is transcriptionally silent (Howard-Till et al. 2013). Thus, a function for Scc2 in gene regulation is unlikely. Therefore, Scc2 in *Tetrahymena* may have lost the cohesin-independent DNA-binding activity and acts primarily to regulate the function of cohesin complexes already bound to chromosomes. The highest levels and strongest localization of Scc2 are found in early prophase of meiosis. Two processes are active in the germline nucleus at that time: DSB induction in preparation for meiotic recombination, and transcription of transposon-like sequences that are destined for elimination from the new somatic nucleus (Mochizuki et al. 2008; Noto and Mochizuki 2017). Although the absence of DSB repair in the Scc2 knockout and knockdown cells suggests an activity in the former process, it is also possible that regulation of cohesin translocation or DNA looping could be involved in the latter.

## Conclusions

We show that Scc3 is not present in the somatic nucleus, which is consistent with the localization of the other cohesin proteins studied to date (Rec8, Smc1, Scc3) (Howard-Till et al. 2013). The absence of cohesin components from the somatic nucleus is somewhat notable, as roles for cohesin in interphase genome organization and gene regulation have almost begun to eclipse its roles in cohesion. However, the radically different organization of *Tetrahymena*’s somatic nucleus may provide some explanation for this. Somatic chromosomes range in size from about 20 kb to 3 Mb (Hamilton et al. 2016), with a median size of 385 kb. In metazoans, Hi-C studies and physical modeling indicate that cohesin mediated looping supports formation of topologically associated domains (TADs) of several hundred kilobase (Gassler et al. 2017; Wutz et al. 2017; Zhan et al. 2017). Therefore, because *Tetrahymena* chromosomes are roughly the size of a TAD, enforcing interactions within a chromosomal domain may be less important than insulating interactions between the small chromosomes. This role may be played by condensin in the somatic nucleus, as recent evidence suggests that condensin is required to evenly distribute chromosome copies throughout the somatic nucleus, as well as prevent general entanglement of chromosomes that disrupts chromosome segregation (Howard-Till and Loidl 2018).

The question remains, however: how is cohesin loaded in *Tetrahymena*? Cohesin is continually present on chromosomes, even after initiation of mitotic or meiotic anaphase (Howard-Till et al. 2013). It is possible that in *Tetrahymena*, cohesin has a bi-modal DNA-binding activity, similar to that shown for condensin (Eeftens et al. 2017). If Scc2 stimulates the ATPase activity of the cohesin ring, this may promote topological entrapment of DNA or otherwise activate the cohesin complex, which is concomitant with Rec8 phosphorylation. In vitro reconstitution experiments using *S. pombe* cohesin have shown that purified cohesin rings composed of Psm1, Psm3, Rad21, and Psc3 (Smc1, Smc3, Scc1, and Scc3) are able to topologically bind DNA in the absence of a loading complex (Murayama and Uhlmann 2014). Therefore, it is also possible the loading complex is not strictly required, but it may enhance the rate of cohesin loading, as suggested by the same study (Murayama and Uhlmann 2014).

The continued lack of evidence for additional cohesin homologs or regulating factors such as Eco1, Pds5, WAPL and Scc4 leads us to believe that, similar to *Tetrahymena*’s use of a stripped down set of recombination proteins (Loidl and Lorenz 2016), the cell has evolved to survive with a minimal set of cohesin factors, as well. Some of this streamlining may be due to a transfer of gene regulation functions to condensin, or a difference in requirements in a polyploid nucleus with small chromosomes. Analysis of Scc3 and Scc2 homologs in *Tetrahymena* has revealed many conserved features between cohesin function in this unusual organism and other model systems. However, many differences still remain, and continued study of cohesin in *Tetrahymena* promises to reveal new insights into the function of this important component of chromosomes.

## Materials and methods

### *Tetrahymena* strains and culture conditions

B2086, CU427, and CU428 strains of *Tetrahymena thermophila* were obtained from the Tetrahymena stock center (Cornell University). These strains were used as wild-type strains. The cells were cultured at 30 °C in Neff’s medium (Orias et al. 1999). Starvation of the cells was performed by incubating the cells in 10 mM Tris-Cl (pH 7.4) for 16 h. Mating was induced by mixing equal numbers (~ 2 × 10^5^ cells/ml) of starved cells from different mating types.

### Protein analysis

For total protein extracts, 100% TCA (*w*/*v*) was added to 5-ml culture to 10% TCA final concentration. Incubation on ice for 1 h was followed by centrifugation at 20,000*g* for 10 min at 4 °C. The pellet was washed three times with cold 100% acetone. The dried pellet was dissolved in 1× sample buffer (50 mM Tris-Cl, pH 6.8, 10% glycerol, 2% SDS, 0.01% bromophenol blue, 5% ß-mercaptoethanol) and used for western blotting analysis.

For immunoprecipitation, cell extracts of specific time points were prepared in lysis buffer (30 mM Tris-Base, 20 mM KCl, 2 mM MgCl_2_, pH 8.1, 150 mM NaCl, 0.1% Triton X-100, 1 mM PMSF, cOmplete protease inhibitor (Roche, Mannheim, Germany)) by using a douncer to break open the cells. The lysate was collected by centrifugation at 25,000 rpm for 50 min at 4 °C, followed by filtration with PVDF filters. Extracts were incubated with magnetic anti-HA beads (Pierce, Rockford, IL, USA) for 2 h at 4 °C. Beads were washed three times with wash buffer (30 mM Tris-Base, 20 mM KCl, 2 mM MgCl_2_, pH 8.1, 150 mM NaCl, 0.1% Triton X-100, cOmplete protease inhibitor (Roche, Mannheim, Germany)). The elution was carried out in 1× sample buffer for 10 min at 95 °C. Mass spectrometry analyses were performed by the MFPL Mass Spectrometry Facility using the VBCF instrument pool. Mass spectrometry data sets are all available in Online Resource 1.

For cell fractionation experiments, cells were incubated in lysis buffer (50 mM Tris pH 8, 100 mM NaCl, 5 mM MgCl2, 1 mM EDTA, 0.5% Triton, Phosphatase and cOmplete Protease Inhibitor (Roche)) for 30 min on ice. The “Soluble” fraction was collected after centrifugation at 15,000*g* for 10 min at 4 °C. The pellet was resuspended in lysis buffer and sonicated four times with 50% power, 5 pulse, 20 s. After centrifugation at 20,000*g* for 10 min at 4 °C, the supernatant was collected as “Chromatin” fraction. Cell fractions were subjected to either TCA precipitation or immunoprecipitation.

Dephosphorylation reactions were performed by incubating bead bound Rec8-HA3His6 from the chromatin fraction of starving cells with lambda protein phosphatase (Lambda PP) (New England BioLabs, Frankfurt, Germany). After IPs, beads were divided into two aliquots and resuspended in 1× Protein MetalloPhosphatase buffer with 1 mM MnCl_2_. One microliter of Lambda PP was added to one aliquot of beads, and both aliquots were incubated 30 min at 30 °C. To stop reactions, beads were washed once in water, then resuspended in sample buffer and boiled 10 min to elute the bound proteins.

### Protein tagging

*SCC3-mCHERRY-NEO4*, *REC8-mCHERRY-NEO4*, *REC8-HA3HIS6-NEO4*, and *SCC2-HA3HIS6-PUR4*, and *SCC3-HA-NEO5* constructs were created by using a C-terminal tagging strategy that expresses the tagged proteins from the endogenous locus (Kataoka et al. 2010). PCR amplification was performed for a 500 bp C-terminal fragment of the gene of interest and a 500 bp fragment downstream of the gene. (See supplemental Table S1 for primers used.) Gibson assembly (NEBuilder HiFi DNA assembly master mix, New England BioLabs) was used to combine PCR fragments with either the *NEO4* or *PUR4* cassettes (gifts from Kazufumi Mochizuki and Takahiko Akematsu) (Kataoka et al. 2010; Iwamoto et al. 2014). The constructs were transformed to the somatic nuclei of different wild-type strains by biolistic transformation as previously described (Bruns and Cassidy-Hanley 1999). The transformants carrying *NEO4*-based constructs were initially selected with 120 μg/ml paromomycin and 0.5 μg/ml CdCl_2_; *NEO5* constructs were selected with 120 μg/ml paromomycin. The paromomycin concentration was gradually increased up to 50 mg/ml while the CdCl_2_ concentration was kept constant at 0.5 μg/ml. For cells transformed with the *PUR*4-based construct, the initial selection was performed with 400 μg/ml puromycin and 0.63 mM CuSO_4_. Puromycin concentration was then increased up to 1200 μg/ml.

### RNAi knockdown

Wild-type genomic DNA was used as a template to PCR amplify a ~ 500 bp fragment of the gene of interest. (See supplemental Table S1 for primer sequences.) The fragments were cloned into the RNAi construct as previously described (Howard-Till et al. 2013). The construct was transformed into either wild-type cells or cells expressing tagged cohesin proteins by biolistic transformation (Bruns and Cassidy-Hanley 1999). Initial selection of the transformants was done by adding 7.5 μg/ml cycloheximide to cells 24 h after bombardment. Selection was performed by increasing the drug concentration up to 30 μg/ml. The expression of the RNA hairpin from the *MTT1* promoter was induced by adding CdCl_2_ to growing (0.5 μg/ml) or starved cultures (0.05 μg/ml) for 24 h.

### Scc2 knockout by co-deletion

Knockout of *SCC2 *was performed using co-deletion as described previously (Hayashi and Mochizuki 2015). Knockouts were confirmed by detection of smaller products in PCR with primers flanking the deleted locus. (See supplemental Table S1 for primer sequences.)

### Immunofluorescence

For localization experiments, 5 ml of cell culture was collected and fixed in 10 mM TRIS pH 7.4 with formaldehyde (4% final concentration) and Triton X-100 (0.5% final concentration) for 30 min at room temperature. The cell suspension was centrifuged, and the pellet was resuspended in 500 μl 4% paraformaldehyde and 3.4% sucrose solution. Eighty microliters of the mixture was spread on a slide and air-dried. The slides were stained with appropriate antibodies and DAPI (4′, 6-diamidino-2-phenylindole). The high-detergent fixation for visualization of chromatin bound proteins was performed by adding a cold mixture of 450 μl of 10% Triton X-100 and 50 μl 37% formaldehyde to 5 ml of cells and incubating for 25 min on ice, then an additional 450 μl of 37% formaldehyde was added to the cells. After 5 min on ice, the cell suspension was centrifuged and resuspended in 500 μl 4% paraformaldehyde and 3.4% sucrose mixture. Eighty microliters of the suspension was spread on a slide and air-dried. Slides for γH2A.X antibody staining were prepared by mixing 5 ml of mating cells with 20 μl of partial Schaudinn’s fixative (saturated HgCl2 and ethanol, 2:1). The cell suspension was incubated at room temperature for 5 min, washed two times with methanol, dropped on a slide, and air-dried. For antibody staining, slides were washed twice with 1× PBS and once with 1× PBS + 0.05% Triton X-100. Primary antibody incubation was performed either at 4 °C over night or at room temperature for 2 h. After washing, secondary antibodies labeled with Cy3 or FITC were applied and incubated at room temperature for 1 h. Slides were washed and mounted with Vectashield anti-fading agent (Vector Laboratories Inc., Burlingame, CA) supplemented with 0.5 μg/ml DAPI.

### Antibodies

The following antibodies were used for western blotting experiments: monoclonal mouse anti-HA (1:1000, Sigma, St. Louis, MO, USA), rabbit polyclonal anti-dsRed (1:1000, Clontech Laboratories, Mountain View, CA, USA), polyclonal rabbit anti-HA (1:1000, Sigma, St. Louis, MO, USA), and monoclonal mouse anti-alpha-tubulin Ab-2 (DM1A) (1:10000, NeoMarkers, Fremont, CA). The following antibodies were used for immunofluorescence analysis: monoclonal mouse anti-HA (1:100, Sigma, St. Louis, MO, USA), rabbit polyclonal anti-dsRed (1:100, Clontech Laboratories, Mountain View, CA, USA), polyclonal rabbit anti-HA (1:100, Sigma, St. Louis, MO, USA), monoclonal mouse anti-Dmc1/Rad51, Clone 51RAD01 (1:50, NeoMarkers, Fremont, CA), and monoclonal mouse anti-phosphorylated H2A.X (1:200, BioLegend, San Diego, CA).

## Electronic supplementary material

Supplemental Table S1 (DOCX 57 kb)

Fig. S1 Scc2 and Rec8 are associated with chromatin in the germline nucleus. Cells expressing Scc2-HA3His6 and Rec8-mCherry were mated with cells expressing only Scc2-HA3His6, and fixed using high detergent to remove unbound proteins from the nucleus. Scc2 is most strongly associated with chromatin in prophase nuclei, whereas Rec8 shows association throughout meiosis and in vegetative cells. Scale bar: 5 μm. (PNG 3246 kb)

High resolution (TIF 11326 kb)

Fig. S2 Gain or loss of chromosomes in *scc2*∆ cells results in abnormal germline nuclei. Examples are shown of a cell with an enlarged germline nucleus and one with a reduced germline nucleus. A WT cell is shown as a comparison. Scale bar: 5 μm. (PNG 254 kb)

High resolution (TIF 578 kb)

Fig. S3 Assessment of cohesion using FISH in meiotic nuclei. **a** Examples of FISH staining in elongated prophase nuclei. In the WT, most nuclei show one or two FISH spots, representing paired or unpaired chromosomes with cohesed chromatids. Upon loss of cohesion, three or four spots can be detected. **b** Western blots of protein extracts prepared from mating cells used in the FISH experiments. In the three cases shown, Scc2-HA3His6 + *scc2*i cells were mated with *scc2*i partners. RNAi was induced for 24 h before starving cells for mating. Protein extracts were prepared from cells 4 h after induction of mating. **c** Plots of FISH data from 4 independent matings of *scc2*i cells, the 3 shown in b as well as a fourth mating cells with *scc2*i in untagged strains. (PNG 233 kb)

High Resolution (TIF 1261 kb)

Online Resource 1 Data sets from mass spectrometry analyses of proteins associated with immunoprecipitated Smc1, Scc2, Scc3, and phospho-peptide identification of Rec8. Tab 1 shows data collected from IPs of Smc1-HA from cell lysates prepared from Smc1-HA cells mated with WT cells collected 4 h and 6 h after initiating mating. Tab. 2, data from IPs of 4 h timepoint of Scc3-HA cells mated with WT. Tab. 3, data from IPs of 4 h timepoint of Scc2-HA3His6 mating cells. Tab. 4, phospho-peptide data from IPs of chromatin fraction of starved cells and soluble fraction of vegetative cells of Rec8-HA3His6. Tab. 5 and Tab. 6 show phospo-peptide data from IPs of vegetative, 0 h, 4 h, 6 h timepoints of Rec8-HA3His6 mating cells. Region 1 and Region 2 are upper and lower regions of the gel used to separate the sample for digestion. Tab. 6 is the biological replicate of the experiment in Tab. 5. For all data sets, protein designations (TTHERM_#) correspond to ID numbers from the Tetrahymena Genome Database (www.ciliate.org). (XLSX 356 kb)
