# Supplementary material for: A streamlined cohesin apparatus is sufficient for mitosis and meiosis in the protist Tetrahymena
Source: Chromosoma. 2018 Jun 12;127(4):421–35. doi: 10.1007/s00412-018-0673-x (PMC6208729; doi:10.1007/s00412-018-0673-x)
Supplement: Supplementary file 1 — (DOCX 57 kb) [file 412_2018_673_MOESM1_ESM.docx]

**Table S1** Primer sequences

| *scc3* RNAi |  |  |
| --- | --- | --- |
|  | apaScc3RNAifor | ATGGGCCCGGAGGCTCTTAAAGAGTTAG |
|  | pmeScc3RNAifor | ATGTTTAAACGGAGGCTCTTAAAGAGTTAG |
|  | xhoScc3RNAirev | ATCTCGAGGGAATCCACTTCATCCAAAAG |
|  | xmaScc3RNAirev | ATCCCGGGGGAATCCACTTCATCCAAAAG |
| *scc2* RNAi |  |  |
|  | 8460_RNAi_apa_FW | CGGGCCCAGTTAGAGGGACAGTATTGAG |
|  | 8460_RNAi_pme_FW | CGTTTAAACAGTTAGAGGGACAGTATTGAG |
|  | 8460_RNAi_sma_RV | GCCCGGGGAATCTTCTCTCTTTCTTATCTG |
|  | 8460_RNAi_xho_RV | GCTCGAGGAATCTTCTCTCTTTCTTATCTG |
| Scc2-HA3His6 |  |  |
|  | 8460_3'_FW | AGGGAACAAAAGCTGGAGCTCGTTCTTCTTACCTTGAAGAAAC |
|  | 8460_3'_RV | CAGGAACATCGTAAGGGTAGGATCCAGCATAGTCATCATCAAAG |
|  | 8460_UTR_FW | ATTTACAGTTAGAGTCGACCTCGAGCAACCTCGCTCCAACTAATTTC |
|  | 8460_UTR_RV | CTATAGGGCGAATTGGGTACCGGTATTGTAGGAATGCTTAGTTG |
| *scc2*Δ |  |  |
|  | Scc2_CDel_FW | TACTCTTCGACCATACTG |
|  | Scc2_CDel_RV | AGTGACTGTCAGAATGAC |
