# Supplementary figures and images for: A streamlined cohesin apparatus is sufficient for mitosis and meiosis in the protist Tetrahymena
Source: Chromosoma. 2018 Jun 12;127(4):421–35. doi: 10.1007/s00412-018-0673-x (PMC6208729; doi:10.1007/s00412-018-0673-x)

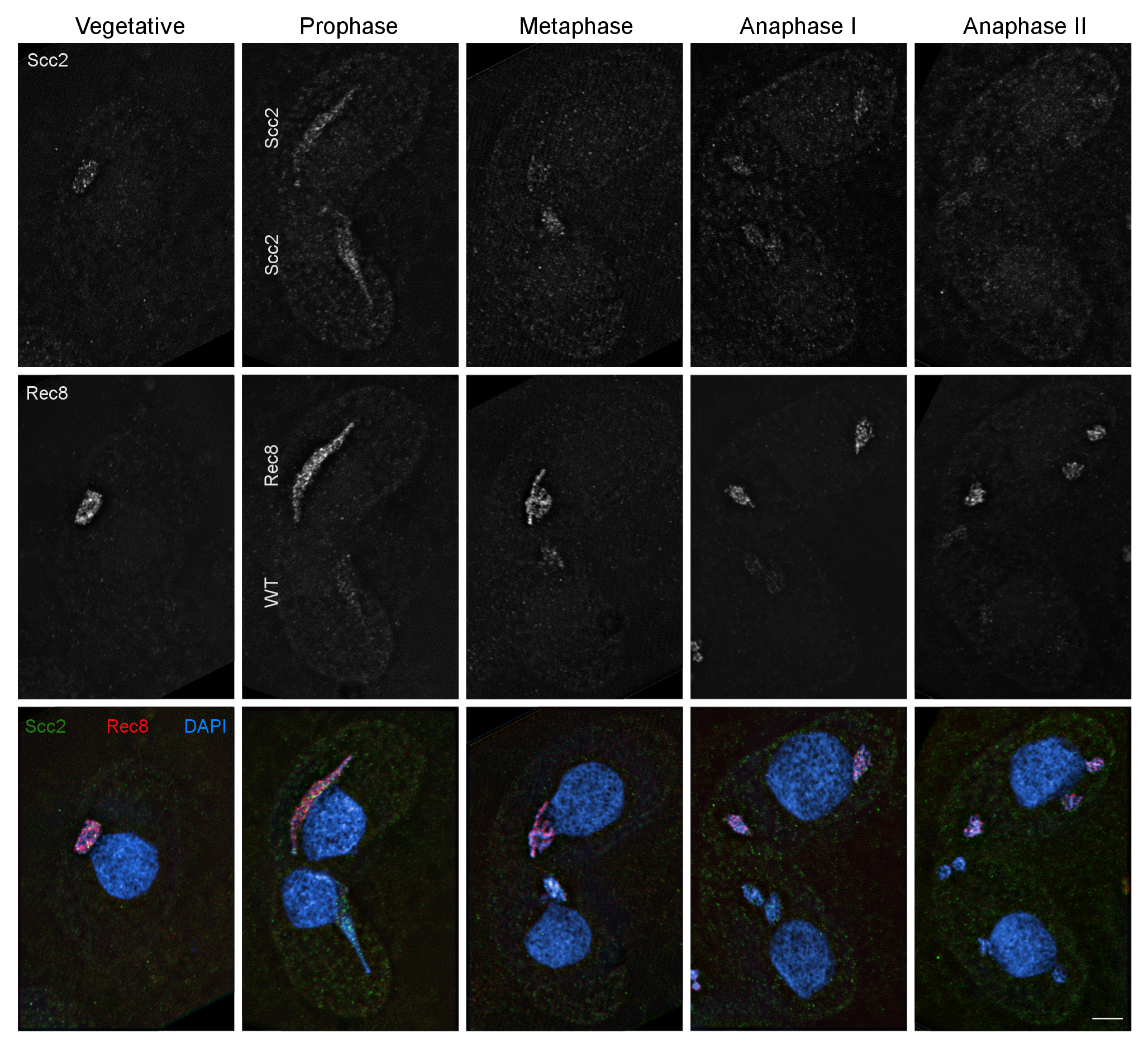

Supplement: Supplementary file 2 — Scc2 and Rec8 are associated with chromatin in the germline nucleus. Cells expressing Scc2-HA3His6 and Rec8-mCherry were mated with cells expressing only Scc2-HA3His6, and fixed using high detergent to remove unbound proteins from the nucleus. Scc2 is most strongly associated with chromatin in prophase nuclei, whereas Rec8 shows association throughout meiosis and in vegetative cells. Scale bar: 5 μm. (PNG 3246 kb) [file 412_2018_673_Fig10_ESM.png]

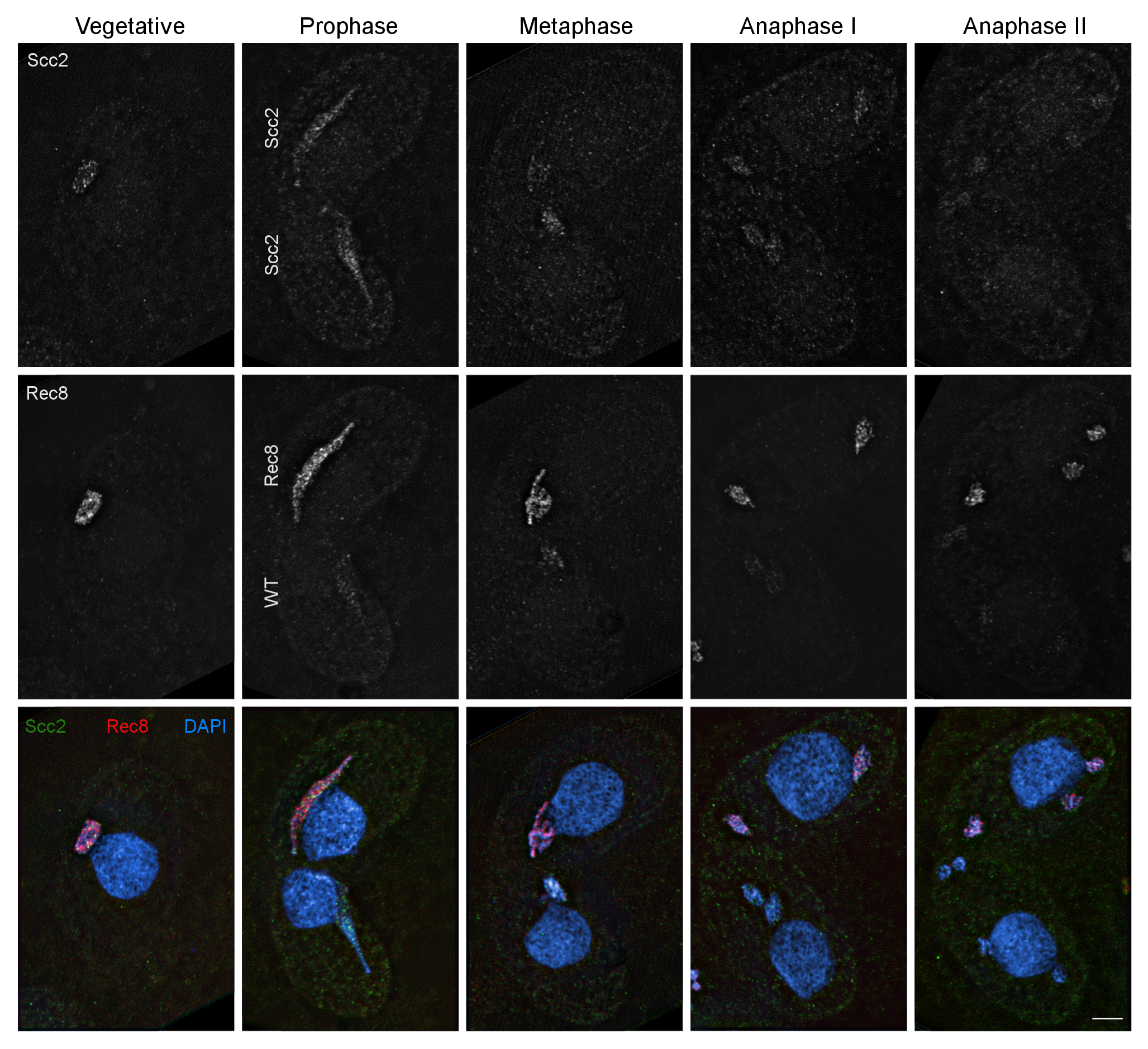

Supplement: Supplementary file 3 — High resolution (TIF 11326 kb) [file 412_2018_673_MOESM2_ESM.tif]

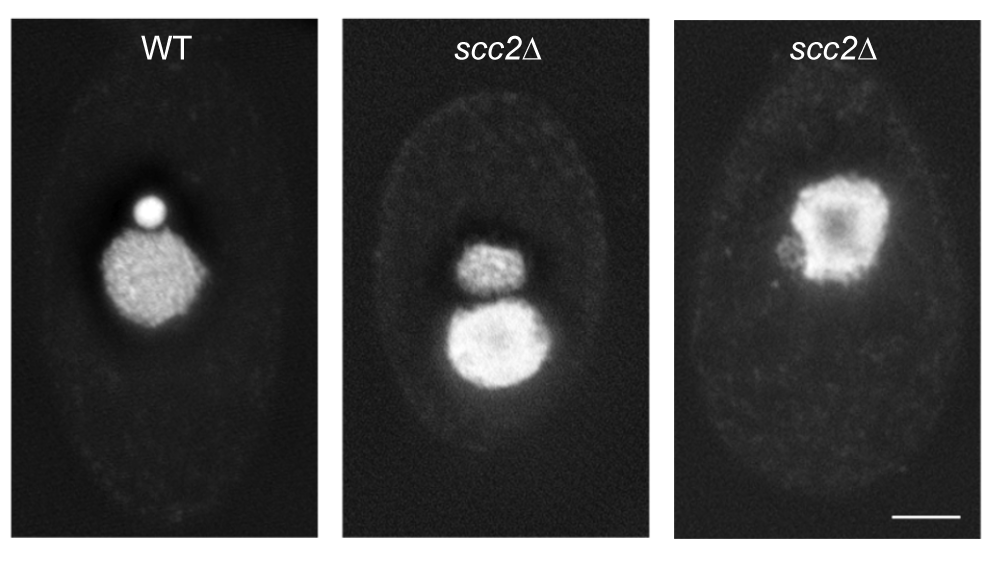

Supplement: Supplementary file 4 — Gain or loss of chromosomes in scc2∆ cells results in abnormal germline nuclei. Examples are shown of a cell with an enlarged germline nucleus and one with a reduced germline nucleus. A WT cell is shown as a comparison. Scale bar: 5 μm. (PNG 254 kb) [file 412_2018_673_Fig11_ESM.png]

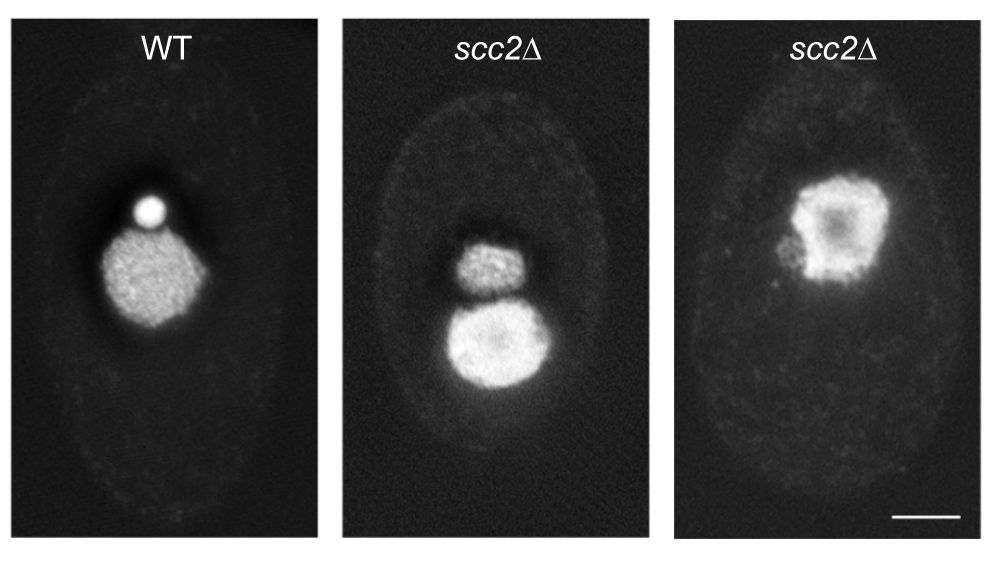

Supplement: Supplementary file 5 — High resolution (TIF 578 kb) [file 412_2018_673_MOESM3_ESM.tif]

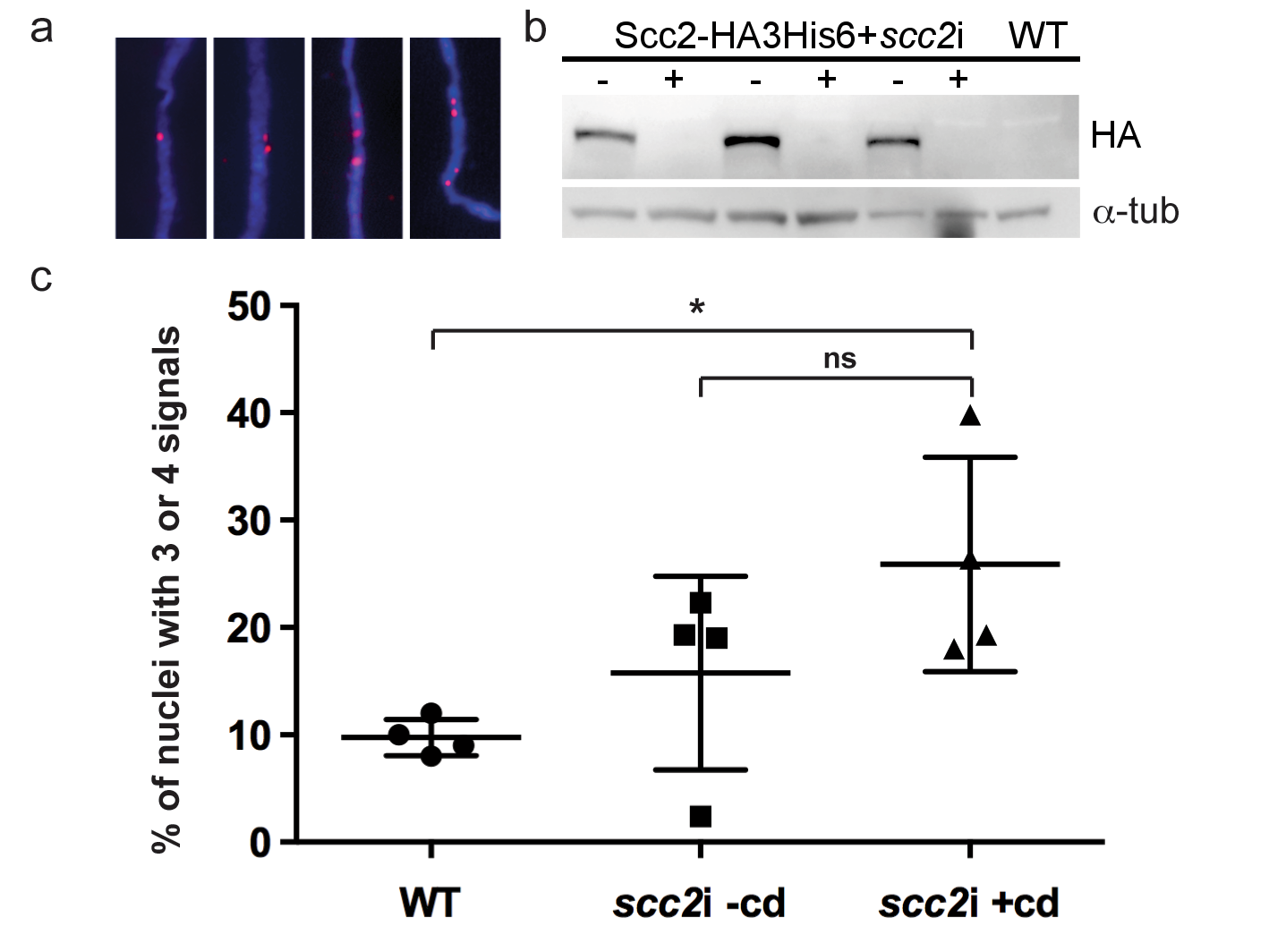

Supplement: Supplementary file 6 — Assessment of cohesion using FISH in meiotic nuclei. a Examples of FISH staining in elongated prophase nuclei. In the WT, most nuclei show one or two FISH spots, representing paired or unpaired chromosomes with cohesed chromatids. Upon loss of cohesion, three or four spots can be detected. b Western blots of protein extracts prepared from mating cells used in the FISH experiments. In the three cases shown, Scc2-HA3His6 + scc2i cells were mated with scc2i partners. RNAi was induced for 24 h before starving cells for mating. Protein extracts were prepared from cells 4 h after induction of mating. c Plots of FISH data from 4 independent matings of scc2i cells, the 3 shown in b as well as a fourth mating cells with scc2i in untagged strains. (PNG 233 kb) [file 412_2018_673_Fig12_ESM.png]

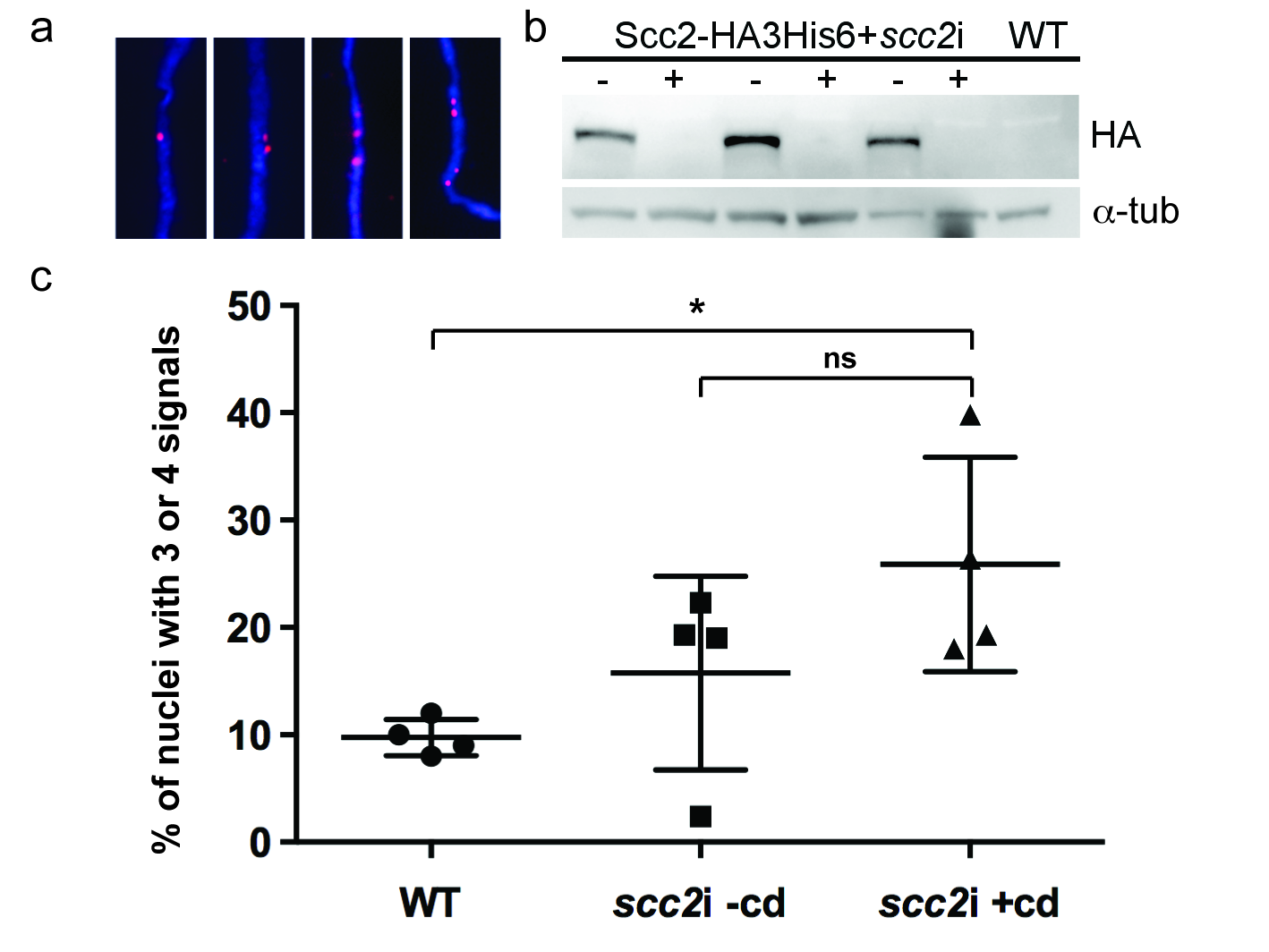

Supplement: Supplementary file 7 — High Resolution (TIF 1261 kb) [file 412_2018_673_MOESM4_ESM.tif]
